# First record of *Phoronopsis* (Phoronida) from Japan, with a description of *Phoronopsiscalifornica* Hilton, 1930 from Okinawa

**DOI:** 10.3897/BDJ.12.e131280

**Published:** 2024-10-08

**Authors:** Masato Hirose, Daisuke Uyeno

**Affiliations:** 1 School of Marine Biosciences, Kitasato University, Kanagawa, Japan School of Marine Biosciences, Kitasato University Kanagawa Japan; 2 Graduate School of Science and Engineering, Kagoshima University, Kagoshima, Japan Graduate School of Science and Engineering, Kagoshima University Kagoshima Japan

**Keywords:** *Phoronopsis*, Phoronida, Okinawa, Japan

## Abstract

**Background:**

Phylum Phoronida currently contains two genera, Phoronis Wright, 1856 and Phoronopsis Gilchrist, 1907, with approximately thirteen speices. Phoronida is distributed worldwide, ranging from northern Europe to southern New Zealand and also from intertidal to 400 m depths. From Japanese waters, four species of *Phoronis* have been reported, viz. *Phoronisijimai* Oka, 1897, *Phoronisaustralis* Haswell, 1883, *Phoronispsammophila* Cori, 1889 and *Phoronisemigi* Hirose et al., 2014.

**New information:**

We describe the morphology of *Phoronopsiscalifornica* Hilton, 1930, based on five specimens collected at a sandy-mud habitat in Nago Bay and Oura Bay, Okinawa, Japan. We examined the internal anatomy by serial paraffin sections. We also examined the genetic distance of Japanese specimens from the other phoronid sequences in GenBank. This is the first record of *Phoronopsis* from Japanese waters and the fifth species record of phoronids in addition to the previously recorded four species of *Phoronis*.

## Introduction

Phoronids, or horseshoe worms, are exclusively marine, sedentary, vermiform animals with a crown of ciliated tentacles, the lophophore, used in suspension feeding. They comprise the small phylum Phoronida, which currently contains two genera, *Phoronis* Wright, 1856 and *Phoronopsis* Gilchrist, 1907, with approximately ten and three species, respectively. *Phoronopsis* is characterised by the presence of an epidermal collar fold at the basis of the lophophore which is absent in *Phoronis*. Phoronid species are also morphologically well defined on the basis of the arrangement and pattern of the body-wall logitudinal musculature, nephridia and lophophore (e.g. Emig (1974), Emig (1979), Emig (1982)).

Phoronida is distributed worldwide, ranging from northern Europe to southern New Zealand and also intertidal to 400 m in depth. From Japanese waters, four species of *Phoronis* have been reported, viz. *Phoronisijimai* Oka, 1897, *Phoronisaustralis* Haswell, 1883, *Phoronispsammophila* Cori, 1889 and *Phoronisemigi* Hirose et al., 2014 (Oka 1897, Hirose et al. 2011, Hirose et al. 2014). In this paper, we report *Phoronopsiscalifornica* Hilton, 1930 for the first time from Japanese waters, with a description of external and internal morphologies and DNA sequences of the nuclear 18S rRNA and the mitochondrial cytochrome *c* oxidase subunit I gene (COI).

## Materials and methods

**Collecting and morphological observation**

**Figure 1.**
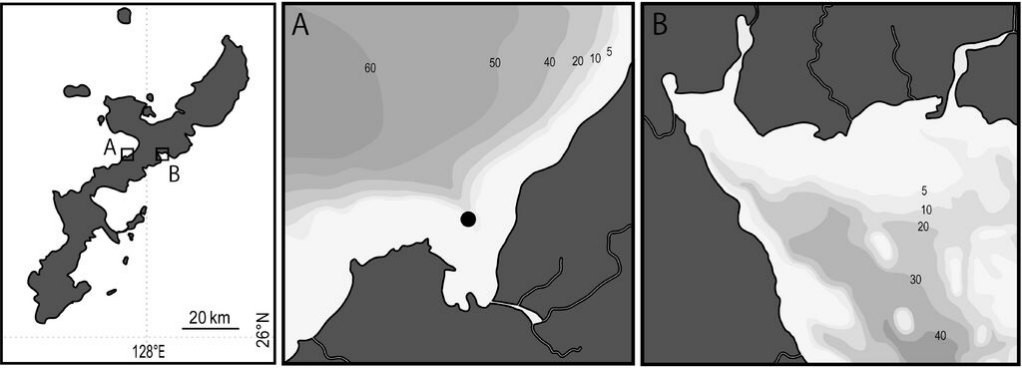
Maps showing the locations of collecting sites.

Specimens of phoronids were collected by SCUBA at Kyoda (26°32'50.61"N, 127°57'41.06"E) in Nago Bay, Okinawa, Japan (Fig. 1) on 22 February 2014. Four specimens were fixed in 10% formalin seawater, but prior to fixation, some tentacles of each individual were also placed directly in 99% ethanol (EtOH) for the following DNA research. We also examined additional specimens collected from Oura Bay, Okinawa, Japan on 27 July 2013.

Measurements of the lophophore and body size were taken from digital photographs with ImageJ 1.37v software (Abramoff et al. 2004). For observation of the internal morphology, four specimens were dehydrated in an ethanol series, cleared in *t*-butanol, embedded in paraffin, sectioned at a thickness of 6 μm and stained with haematoxylin-eosin (HE). Volume Extractor 3.0 software (i-Plants Systems) was used to construct three-dimensional images of the nephridium. All specimens have been deposited in the National Museum of Nature and Science, Tsukuba, Japan (NSMT).

**DNA extraction and PCR amplification**

Total genomic DNA was extracted from the tentacles of two of ethanol-fixed specimens of different colour (NSMT-Te 1031 and 1032), using a DNeasy Blood and Tissue Kit (Qiagen), following the manufacturer’s protocol. The 18S gene was amplified with three primer sets: 1F/4R, 1F/1270R, 3F/18sbi and 18Sa2.0/9R (Giribet et al. 1996, Whiting et al. 1997, Littlewood and Olson 2001). The COI fragment was amplified with the primer pair LCO1490/HCO2198 (Folmer et al. 1994). PCR reactions were performed with *ExTaq* (TaKaRa). Conditions for hot-start thermal cycling were 4 min at 94°C; 40 cycles of 30 sec at 94°C, 30 sec at 50–52°C and 90 sec at 72°C; and 7 min at 72°C. PCR products were visualised on a 1% agarose gel and purified according to the method of Boom et al. (1990) with some modifications (Kobayashi and Tachi 2009, Kobayashi et al. 2009). Cycle sequencing was performed with BigDye Terminator 3.1 (Life Technologies). Both product strands were sequenced with an ABI 3130 Genetic Analyzer (Life Technologies). Chromatograms were edited and overlapping sequence fragments were assembled using MEGA 6.06 (Tamura et al. 2013). The sequences have been deposited with DDBJ/EMBL/GenBank under accession numbers LC835677–LC835680 for the two specimens of *Phoronopsiscalifornica* Hilton, 1930 from Okinawa (Table 1).

**Molecular phylogeny**

The 18S and COI sequences obtained for *Phoronopsiscalifornica* were aligned with those from other bryozoans deposited in GenBank (Table 1) using Clustal W (Thompson et al. 1994) implemented in MEGA 6.06 (Tamura et al. 2013). The alignment was performed gene by gene. Most of the sites for both 18S and COI were unambiguously aligned. We therefore used the entire region, excluding gap sites, for our phylogenetic analyses.

Maximum Likelihood (ML) analyses were performed with MEGA 6.06. The best-fit model for the 18S and COI sequences determined by the AICc implemented in MEGA 6.06 were TN93+G (Tamura-Nei model [Tamura and Nei 1993] with gamma-distributed rates) and GTR+G+I (general time reversible [Tavaré 1986] with gamma-distributed rates and invariant rates amongst sites), respectively. Optimal ML trees were found by a tree-bisection reconnection (TBR) search, starting with a tree topology generated by the BIONJ method (Gascuel 1997) using Maximum Composite Likelihood (MCL) distances (Tamura et al. 2004). One-thousand bootstrap pseudo-replicates were analysed to obtain nodal support values. The trees were rooted with some brachiopods (*Glottidiapyramidata*, *Novocraniaanomala* and Disciniscacf.tenuis) and *Phoronisovalis* Wright, 1856, based on the results of previous studies, respectively (Halanych et al. 1995, Cohen and Weydmann 2005, Santagata and Cohen 2009).

Since most of the sequences used in this study were obtained from GenBank, we used the original specific names in GenBank given by previous authors (Halanych et al. 1995, Cohen et al. 1998, Cohen 2000, Giribet et al. 2000, Mallatt and Winchell 2002, Helfenbein and Boore 2004, Cohen and Weydmann 2005, Santagata and Cohen 2009) and followed with the taxonomically valid specific names if necessary.

## Data resources

All specimens have been deposited in the National Museum of Nature and Science, Tsukuba, Japan (NSMT) under the catalogue numbers NSMT-Te1028–1040. The sequences have been deposited with DDBJ/EMBL/GenBank under accession numbers LC835677–LC835680 for the two specimens of *Phoronopsiscalifornica* Hilton, 1930 from Okinawa (Table 1).

## Taxon treatments

### Phoronopsis californica

Hilton, 1930

4292D897-9129-52EF-A59F-CBB3F6910966

*Phoronopsiscalifornica* Hilton, 1930 - Hilton (1930), 154–158; Emig (1969), 479–482, Figs. 10–13; Emig (1971), 554–555, Pl. 3, Figs. 8–9, Pl. 6, Figs. 5–8, Pl. 7, Fig. 11; Emig (1973), 17–20, Figs. 8g–m, 9, 10; Temereva et al. (2016), 521, Fig. 32.

#### Materials

- **Type status:** Other material. **Occurrence:** catalogNumber: NSMT-Te 1028; recordedBy: Masato Hirose, Daisuke Uyeno; individualCount: 1; lifeStage: adult; preparations: preserved in 70% ethanol; disposition: in collection; occurrenceID: 54CFF287-778C-5937-AE70-1837CA5DDC26; **Taxon:** scientificName: Phoronopsiscalifornica Hilton, 1930; kingdom: Animalia; phylum: Phoronida; genus: Phoronopsis; specificEpithet: californica; scientificNameAuthorship: Hilton, 1930; nomenclaturalCode: ICZN; **Location:** country: Japan; stateProvince: Okinawa; locality: Oura Bay, Okinawa, Japan; verbatimDepth: 6 m; **Identification:** identifiedBy: Masato Hirose; **Event:** samplingProtocol: SCUBA; eventDate: 27-07-2013; **Record Level:** language: en; ownerInstitutionCode: NSMT; basisOfRecord: PreservedSpecimen; informationWithheld: single upper part of individual including lophophore
- **Type status:** Other material. **Occurrence:** catalogNumber: NSMT-Te 1029; recordedBy: Masato Hirose, Daisuke Uyeno; individualCount: 1; lifeStage: adult; preparations: fixed with 10% formalin and preserved in 70% ethanol; disposition: in collection; occurrenceID: 3DF81EA3-A31B-53C8-A1CA-8038C8B04CFB; **Taxon:** scientificName: Phoronopsiscalifornica Hilton, 1930; kingdom: Animalia; phylum: Phoronida; genus: Phoronopsis; specificEpithet: californica; scientificNameAuthorship: Hilton, 1930; nomenclaturalCode: ICZN; **Location:** country: Japan; stateProvince: Okinawa; locality: Kyoda, Nago Bay, Okinawa, Japan; verbatimDepth: 5–10 m; verbatimCoordinates: 26°32'50.61"N, 127°57'41.06"E; **Identification:** identifiedBy: Masato Hirose; **Event:** samplingProtocol: SCUBA; eventDate: 22-02-2014; **Record Level:** language: en; ownerInstitutionCode: NSMT; basisOfRecord: PreservedSpecimen; informationWithheld: individual
- **Type status:** Other material. **Occurrence:** catalogNumber: NSMT-Te 1030; recordedBy: Masato Hirose, Daisuke Uyeno; individualCount: 1; lifeStage: adult; preparations: fixed with 10% formalin and preserved in 70% ethanol; disposition: in collection; occurrenceID: 49663507-1E6B-5DFC-AA2C-8125A022A320; **Taxon:** scientificName: Phoronopsiscalifornica Hilton, 1930; kingdom: Animalia; phylum: Phoronida; genus: Phoronopsis; specificEpithet: californica; scientificNameAuthorship: Hilton, 1930; nomenclaturalCode: ICZN; **Location:** country: Japan; stateProvince: Okinawa; locality: Kyoda, Nago Bay, Okinawa, Japan; verbatimDepth: 5–10 m; verbatimCoordinates: 26°32'50.61"N, 127°57'41.06"E; **Identification:** identifiedBy: Masato Hirose; **Event:** samplingProtocol: SCUBA; eventDate: 22-02-2014; **Record Level:** language: en; ownerInstitutionCode: NSMT; basisOfRecord: PreservedSpecimen; informationWithheld: individuals
- **Type status:** Other material. **Occurrence:** catalogNumber: NSMT-Te 1031; recordedBy: Masato Hirose, Daisuke Uyeno; individualCount: 1; lifeStage: adult; preparations: fixed and preserved in 10% formalin; disposition: in collection; occurrenceID: 94EE2857-5F54-5237-947E-30EFB70DDEA1; **Taxon:** scientificName: Phoronopsiscalifornica Hilton, 1930; kingdom: Animalia; phylum: Phoronida; genus: Phoronopsis; specificEpithet: californica; scientificNameAuthorship: Hilton, 1930; nomenclaturalCode: ICZN; **Location:** country: Japan; stateProvince: Okinawa; locality: Kyoda, Nago Bay, Okinawa, Japan; verbatimDepth: 5–10 m; verbatimCoordinates: 26°32'50.61"N, 127°57'41.06"E; **Identification:** identifiedBy: Masato Hirose; **Event:** samplingProtocol: SCUBA; eventDate: 22-02-2014; **Record Level:** language: en; ownerInstitutionCode: NSMT; basisOfRecord: PreservedSpecimen; informationWithheld: dissected individual
- **Type status:** Other material. **Occurrence:** catalogNumber: NSMT-Te 1032; recordedBy: Masato Hirose, Daisuke Uyeno; individualCount: 1; lifeStage: adult; preparations: fixed and preserved in 10% formalin; disposition: in collection; occurrenceID: 13C7149E-F548-5FD0-B58A-1BAF572C526F; **Taxon:** scientificName: Phoronopsiscalifornica Hilton, 1930; kingdom: Animalia; phylum: Phoronida; genus: Phoronopsis; specificEpithet: californica; scientificNameAuthorship: Hilton, 1930; nomenclaturalCode: ICZN; **Location:** country: Japan; stateProvince: Okinawa; locality: Kyoda, Nago Bay, Okinawa, Japan; verbatimDepth: 5–10 m; verbatimCoordinates: 26°32'50.61"N, 127°57'41.06"E; **Identification:** identifiedBy: Masato Hirose; **Event:** samplingProtocol: SCUBA; eventDate: 22-02-2014; **Record Level:** language: en; ownerInstitutionCode: NSMT; basisOfRecord: PreservedSpecimen; informationWithheld: dissected individual
- **Type status:** Other material. **Occurrence:** catalogNumber: NSMT-Te 1033; recordedBy: Masato Hirose, Daisuke Uyeno; individualCount: 1; preparations: fixed and preserved in 99% ethanol; disposition: in collection; occurrenceID: 006A899A-3AA4-5917-AD27-364A5FC6ADDC; **Taxon:** scientificName: Phoronopsiscalifornica Hilton, 1930; kingdom: Animalia; phylum: Phoronida; genus: Phoronopsis; specificEpithet: californica; scientificNameAuthorship: Hilton, 1930; nomenclaturalCode: ICZN; **Location:** country: Japan; stateProvince: Okinawa; locality: Kyoda, Nago Bay, Okinawa, Japan; verbatimDepth: 5–10 m; verbatimCoordinates: 26°32'50.61"N, 127°57'41.06"E; **Identification:** identifiedBy: Masato Hirose; **Event:** samplingProtocol: SCUBA; eventDate: 22-02-2014; **Record Level:** language: en; ownerInstitutionCode: NSMT; basisOfRecord: PreservedSpecimen; informationWithheld: empty tube
- **Type status:** Other material. **Occurrence:** catalogNumber: NSMT-Te 1034; recordedBy: Masato Hirose, Daisuke Uyeno; individualCount: 1; preparations: fixed and preserved in 99% ethanol; disposition: in collection; occurrenceID: 25D80D46-5EB8-5B3F-8A67-81D8D84D8BCA; **Taxon:** scientificName: Phoronopsiscalifornica Hilton, 1930; kingdom: Animalia; phylum: Phoronida; genus: Phoronopsis; specificEpithet: californica; scientificNameAuthorship: Hilton, 1930; nomenclaturalCode: ICZN; **Location:** country: Japan; stateProvince: Okinawa; locality: Kyoda, Nago Bay, Okinawa, Japan; verbatimDepth: 5–10 m; verbatimCoordinates: 26°32'50.61"N, 127°57'41.06"E; **Identification:** identifiedBy: Masato Hirose; **Event:** samplingProtocol: SCUBA; eventDate: 22-02-2014; **Record Level:** language: en; ownerInstitutionCode: NSMT; basisOfRecord: PreservedSpecimen; informationWithheld: empty tube
- **Type status:** Other material. **Occurrence:** catalogNumber: NSMT-Te 1035; recordedBy: Masato Hirose, Daisuke Uyeno; individualCount: 1; preparations: fixed and preserved in 99% ethanol; disposition: in collection; occurrenceID: 2F908976-6845-5C0D-809A-31957348DDE7; **Taxon:** scientificName: Phoronopsiscalifornica Hilton, 1930; kingdom: Animalia; phylum: Phoronida; genus: Phoronopsis; specificEpithet: californica; scientificNameAuthorship: Hilton, 1930; nomenclaturalCode: ICZN; **Location:** country: Japan; stateProvince: Okinawa; locality: Kyoda, Nago Bay, Okinawa, Japan; verbatimDepth: 5–10 m; verbatimCoordinates: 26°32'50.61"N, 127°57'41.06"E; **Identification:** identifiedBy: Masato Hirose; **Event:** samplingProtocol: SCUBA; eventDate: 22-02-2014; **Record Level:** language: en; ownerInstitutionCode: NSMT; basisOfRecord: PreservedSpecimen; informationWithheld: empty tube
- **Type status:** Other material. **Occurrence:** catalogNumber: NSMT-Te 1036; recordedBy: Masato Hirose, Daisuke Uyeno; individualCount: 1; preparations: fixed and preserved in 99% ethanol; disposition: in collection; occurrenceID: AD4B02A4-4761-5198-8FE7-EE51981D6580; **Taxon:** scientificName: Phoronopsiscalifornica Hilton, 1930; kingdom: Animalia; phylum: Phoronida; genus: Phoronopsis; specificEpithet: californica; scientificNameAuthorship: Hilton, 1930; nomenclaturalCode: ICZN; **Location:** country: Japan; stateProvince: Okinawa; locality: Kyoda, Nago Bay, Okinawa, Japan; verbatimDepth: 5–10 m; verbatimCoordinates: 26°32'50.61"N, 127°57'41.06"E; **Identification:** identifiedBy: Masato Hirose; **Event:** samplingProtocol: SCUBA; eventDate: 22-02-2014; **Record Level:** language: en; ownerInstitutionCode: NSMT; basisOfRecord: PreservedSpecimen; informationWithheld: empty tube
- **Type status:** Other material. **Occurrence:** catalogNumber: NSMT-Te 1037; recordedBy: Masato Hirose, Daisuke Uyeno; individualCount: 1; lifeStage: adult; preparations: 20 slides of 6-μm transverse sections stained with HE; disposition: in collection; occurrenceID: 83B05256-0FC6-536E-B8A0-D1E5E2F84A2C; **Taxon:** scientificName: Phoronopsiscalifornica Hilton, 1930; kingdom: Animalia; phylum: Phoronida; genus: Phoronopsis; specificEpithet: californica; scientificNameAuthorship: Hilton, 1930; nomenclaturalCode: ICZN; **Location:** country: Japan; stateProvince: Okinawa; locality: Kyoda, Nago Bay, Okinawa, Japan; verbatimDepth: 5–10 m; verbatimCoordinates: 26°32'50.61"N, 127°57'41.06"E; **Identification:** identifiedBy: Masato Hirose; **Event:** samplingProtocol: SCUBA; eventDate: 22-02-2014; **Record Level:** language: en; ownerInstitutionCode: NSMT; basisOfRecord: PreservedSpecimen
- **Type status:** Other material. **Occurrence:** catalogNumber: NSMT-Te 1038; recordedBy: Masato Hirose, Daisuke Uyeno; individualCount: 1; lifeStage: adult; preparations: 8 slides of 6-μm transverse sections stained with HE; disposition: in collection; occurrenceID: F07C5C9E-22DF-543B-9AD3-75A21EB0B9AC; **Taxon:** scientificName: Phoronopsiscalifornica Hilton, 1930; kingdom: Animalia; phylum: Phoronida; genus: Phoronopsis; specificEpithet: californica; scientificNameAuthorship: Hilton, 1930; nomenclaturalCode: ICZN; **Location:** country: Japan; stateProvince: Okinawa; locality: Kyoda, Nago Bay, Okinawa, Japan; verbatimDepth: 5–10 m; verbatimCoordinates: 26°32'50.61"N, 127°57'41.06"E; **Identification:** identifiedBy: Masato Hirose; **Event:** samplingProtocol: SCUBA; eventDate: 22-02-2014; **Record Level:** language: en; ownerInstitutionCode: NSMT; basisOfRecord: PreservedSpecimen
- **Type status:** Other material. **Occurrence:** catalogNumber: NSMT-Te 1039; recordedBy: Masato Hirose, Daisuke Uyeno; individualCount: 1; lifeStage: adult; preparations: 16 slides of 6-μm longitudinal sections stained with HE; disposition: in collection; occurrenceID: A7819A85-F858-53DB-BEA6-4D6DEB53DC72; **Taxon:** scientificName: Phoronopsiscalifornica Hilton, 1930; kingdom: Animalia; phylum: Phoronida; genus: Phoronopsis; specificEpithet: californica; scientificNameAuthorship: Hilton, 1930; nomenclaturalCode: ICZN; **Location:** country: Japan; stateProvince: Okinawa; locality: Kyoda, Nago Bay, Okinawa, Japan; verbatimDepth: 5–10 m; verbatimCoordinates: 26°32'50.61"N, 127°57'41.06"E; **Identification:** identifiedBy: Masato Hirose; **Event:** samplingProtocol: SCUBA; eventDate: 22-02-2014; **Record Level:** language: en; ownerInstitutionCode: NSMT; basisOfRecord: PreservedSpecimen
- **Type status:** Other material. **Occurrence:** catalogNumber: NSMT-Te 1040; recordedBy: Masato Hirose, Daisuke Uyeno; individualCount: 1; lifeStage: adult; preparations: 20 slides of 6-μm longitudinal sections stained with HE; disposition: in collection; occurrenceID: DDA70FBC-2DBF-5280-BE54-A66B3D194BAA; **Taxon:** scientificName: Phoronopsiscalifornica Hilton, 1930; kingdom: Animalia; phylum: Phoronida; genus: Phoronopsis; specificEpithet: californica; scientificNameAuthorship: Hilton, 1930; nomenclaturalCode: ICZN; **Location:** country: Japan; stateProvince: Okinawa; locality: Kyoda, Nago Bay, Okinawa, Japan; verbatimDepth: 5–10 m; verbatimCoordinates: 26°32'50.61"N, 127°57'41.06"E; **Identification:** identifiedBy: Masato Hirose; **Event:** samplingProtocol: SCUBA; eventDate: 22-02-2014; **Record Level:** language: en; ownerInstitutionCode: NSMT; basisOfRecord: PreservedSpecimen

#### Description

**Figure 2.**
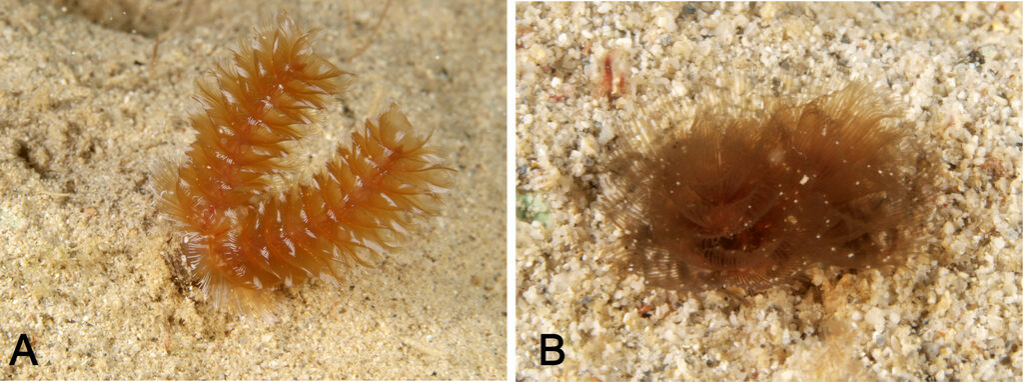
*Phoronopsiscalifornica* Hilton, 1930. **A** Living adult individual found in Nago Bay (NSMT-Te1029); **B** living juvenile individual with dark colour lophophore found in Nago Bay (NSMT-Te1031).

**Figure 3.**
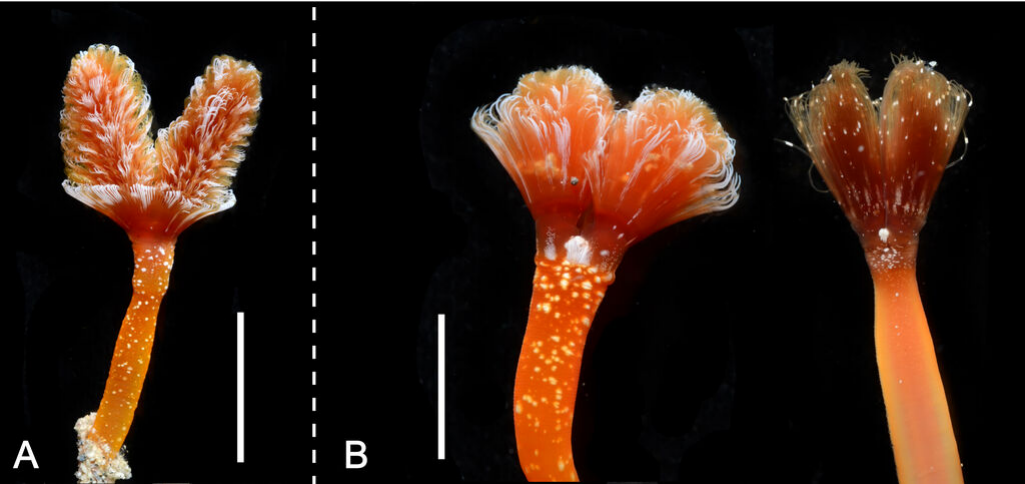
*Phoronopsiscalifornica* Hilton, 1930. **A** Oral view of anterior part of the body of living adult individual (NSMT-Te1029); **B** anal view of anterior part of two juvenile individuals of different colour; both showing white pigmented anal papilla (NSMT-Te1032, 1031). Scale bars: A = 1 cm; B = 5 mm.

**Figure 4.**
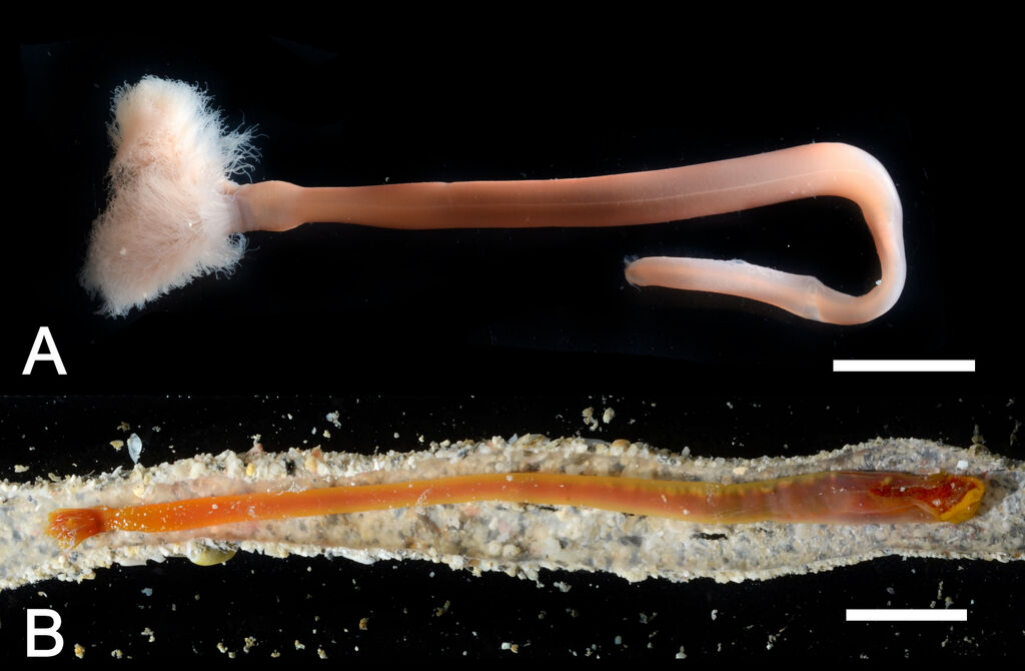
*Phoronopsiscalifornica* Hilton, 1930. **A** Preserved individual (10% formalin seawater); **B** living individual in a dissected tube. Scale bars: 1 cm.

**Figure 5.**
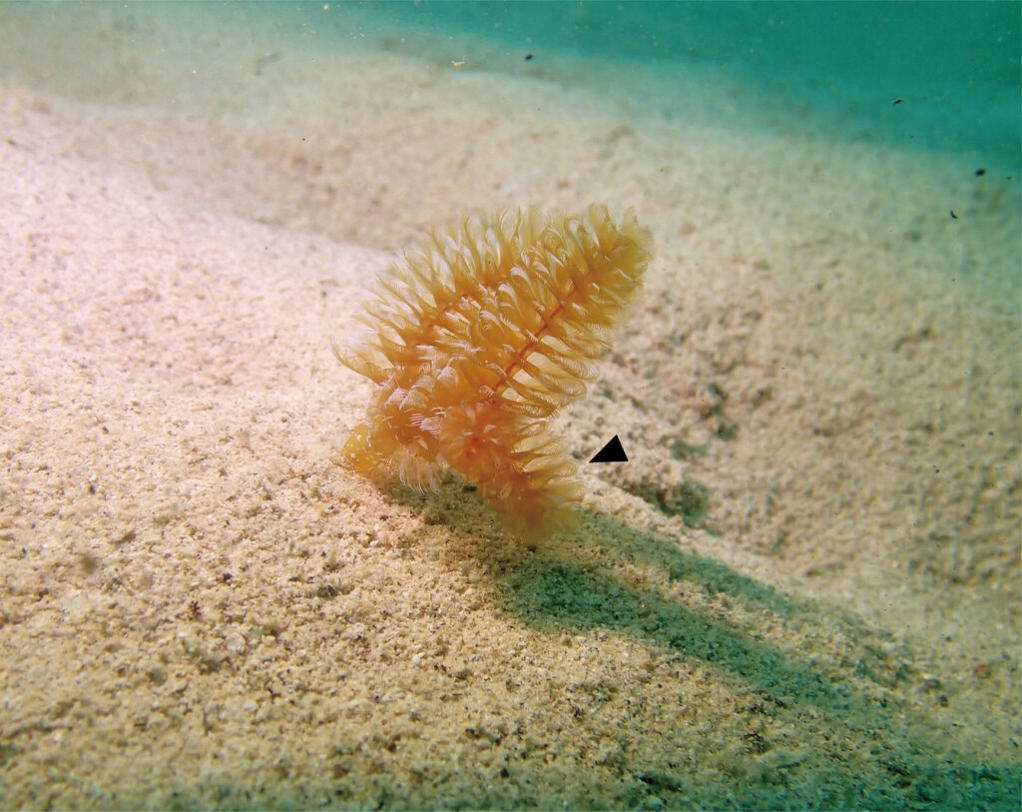
*Phoronopsiscalifornica* Hilton, 1930, NSMT-Te 1030, bearing additional arm of lophophore (black arrowhead).

Body, except lophophore 27.62–116.56 mm in length (avg. 73.85 ± 31.52 mm, n = 5) in contracted state; 2.79–4.58 mm in diameter at ampula (avg. 3.77 ± 0.91 mm, n = 3); other parts ranged 1.25–3.90 mm in diameter; orange or brown with white spots in living state (Figs 2, 3), yellowish-white after fixation (Fig. 4A). Lophophore spirally-shaped in juvenile and helicoidal-shaped in adult with 6–10 coils on each side (Fig. 4); 5.13–15.41 mm in length (avg. 9.48 ± 4.04 mm, n = 7), 1.82–3.40 mm in diameter basally (avg. 2.60 ± 0.61 mm, n = 7); tentacles more than 1600 in number; orange or brown with white spots in life. Inhabits a cylindrical tube constructed with small sand grains (Fig. 4B); 75.43–209.70 mm in length (avg. 119.52 ± 52.77 mm, n = 5); 7.00–15.41 mm in diameter (avg. 7.88 ± 0.56 mm, n = 5). Single individual observed bearing an additional arm of lophophore was considered a morphological malformation (Fig. 5). Anal papilla densely covered with white pigments (Fig. 3B).

**Figure 6.**
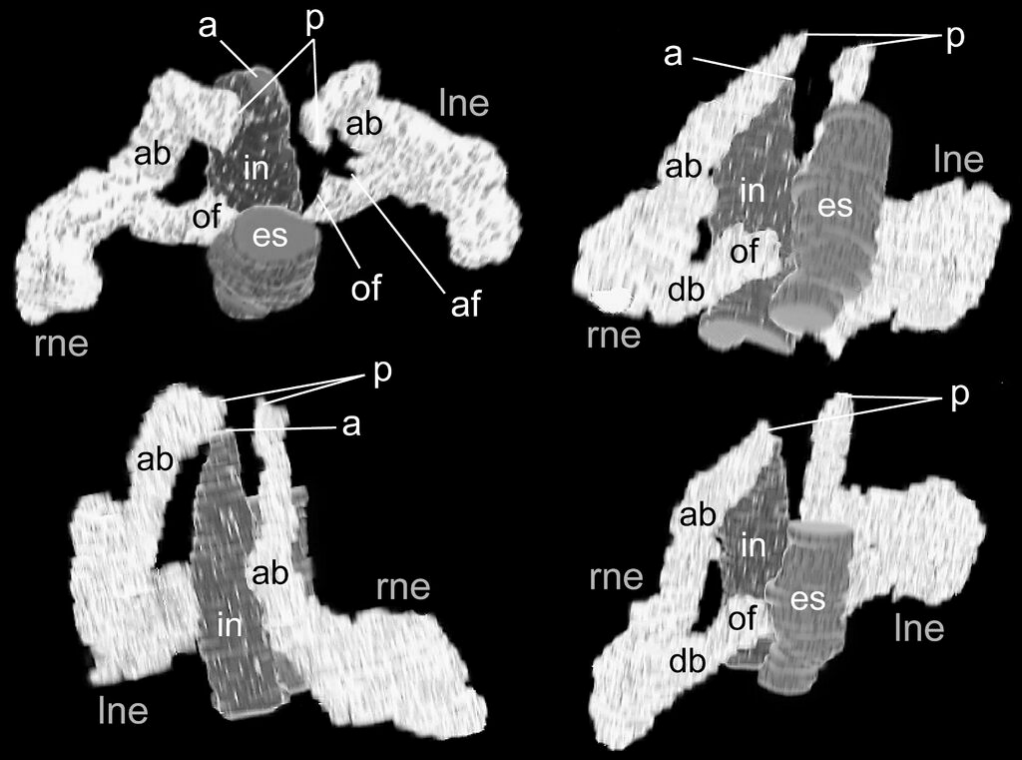
Reconstructed three-dimensional images of the nephridia of *Phoronopsiscalifornica* Hilton, 1930, from NSMT-Te 1037 showing the long curved ascending branch along the intestine and large nephridial (anal) funnels extending towards the osophagus. Abbreviations: a, anus; ab, ascending branch; af, anal funnel; db, descending branch; es, oesophagus; in, intestine; lne, left nephridium; of, oral funnel; p, nephridiopore; rne, right nephridium.

**Figure 7.**
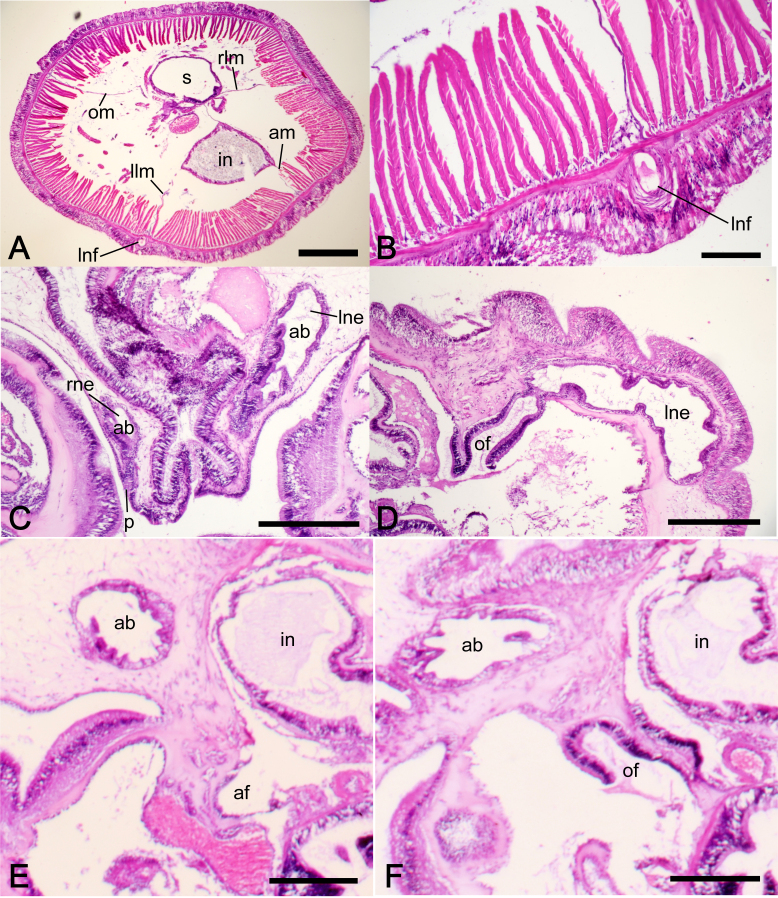
*Phoronopsiscalifornica* Hilton, 1930. **A** transverse section through the posterior part of the body, showing four mesenteries and the position of the giant nerve fibre, NSMT-Te 1038; **B** enlargement of transverse section showing longitudinal muscles and giant nerve fibre, NSMT-Te 1038; **C** transverse section through the basal part of lophophore showing top of the ascending branches of the nerphridia with the nephridiopore on the invagination of the collar fold, NSMT-Te 1037; **D** transverse section through the nephridium showing the nephridial funnel (oral funnel), NSMT-Te 1037; **E** upper part of nephridial funnel showing anal funnel, NSMT-Te 1037; **F** lower part of nephridial funnel showing oral funnel, NSMT-Te 1037. Abbreviations: ab, ascending branch; af, anal funnel; am, anal mesentery; in, intestine; llm, left lateral mesentery; lne, left nephridium; lnf, left giant nerve fibre; of, oral funnel; om, oral mesentery; p, nephridiopore; rlm, right lateral mesentery; rne, right nephridium; s, stomach. Scale bars: A = 500 μm; B = 100 μm; C, D = 300 μm; E, F = 200 μm.

Nephridia 762–888 μm in height (avg. 825 ± 89 μm, n = 2), with long slightly curved ascending branch (ab) and short descending branch (db) (Fig. 6), ab/db length ratio 2.6 (n = 2). Nephridial pore situated same or slightly above the anus level on the invagination of collar fold (Fig. 6), opening against intestine, about 24 μm in diameter (Fig. 7C). Ascending branch located near intestine, lying on the body axis, slightly curved along the body wall (Fig. 6); 648–888 μm in height (avg. 768 ± 170 μm, n = 2). Descending branch situated the lower part of the nephridium, on the oesophagus side of ascending branch; 234–462 μm in height (avg. 348 ± 161 μm, n = 2). Two nephridial funnels present; anal funnel slightly larger than oral funnel in height; 126–156 μm (avg. 141 ± 21 μm, n = 2) and 102–162 μm (avg. 132 ± 42 μm, n = 2), respectively. The aperture of nephridial funnels located on lateral surface of upper part of descending branch; the anal funnel faces intestine, while the oral funnel faces oesophagus (Figs 6, 7D–F). Oral funnel situated slightly lower than anal funnel.

Body-wall longitudinal muscles of feathery type (Fig. 7A and 7B); 226–262 in number, arranged as in the following formula (Selys-Longchamps 1907):

Composite formula Mean formula

[226–262] \begin{varwidth}{50in}\begin{equation*}
            65-88|74-81\over45-53|42-45
        \end{equation*}\end{varwidth} 243.5 = \begin{varwidth}{50in}\begin{equation*}
            72.5|78.0\over49.0|44.0
        \end{equation*}\end{varwidth} (n = 4 sections from 2 individuals)

Left and right lateral mesenteries present (Fig. 7A). Single left giant nerve fibre present; transversally oblong, 62.0–72.0 μm wide and 92.9–123.5 μm long (avg. 670 ± 7.1 μm and 108.2 ± 21.6 μm, respectively, based on two sections from different parts of the body, from single individual), situated at the base of left lateral mesentery below the nephridial level (Fig. 7B).

Gonads not observed in any of our specimens; sex could, thus, not be determined.

#### Distribution

*Phoronopsiscalifornica* was originally described from the Balboa Bay, Los Angeles in USA and distributed along the west coasts of North America, coast of Spain, Madagascar, Mozambique Channel and the South China Sea (Emig 1969, Emig and Plante 1969, Emig 1971, Emig 1973, Thomassin and Emig 1983, Sánchez Tocino et al. 1997, Temereva 2009, Temereva et al. 2016). From Japan, this species is known only from a soft sandy-mud bottom in the inner area of Oura Bay and Nago Bay, Okinawa.

#### Taxon discussion

Our material of *Phoronopsiscalifornica* collected from Okinawa agrees with previous morphological accounts of this species (Hilton 1930, Emig 1969, Emig 1971, Emig 1973) in the following characters: 1) the large helicoidal-shaped lophophore with bright colours in adult individuals, 2) nephridium with the long ascending branch and two nephridial funnels on the descending branch and 3) the number of longitudinal muscles in the left anal and both oral coeloms. All of our specimens have a remarkably shorter body length compared to that of *P.californica* (220–450 mm) previously reported (Emig 1979), which is probably due to the contracted state and/or incomplete specimens. Some of our specimens are spirally-shaped; those specimens were probably a juvenile. Our specimens have a slightly larger number of longitudinal muscles in the right anal coeloms compared to the previous descriptions and the difference of height of oral and anal nephridial funnels are relatively smaller. Temereva et al. (2016) reported *P.californica* from the South China Sea; however, the identification is only based on the underwater photograph. This is the first record of the internal morphology and molecular data of *P.californica* from the north-western Pacific.

## Analysis

In this study, most of the sites for both 18S and COI were unambiguously aligned; therefore, we used the entire region excluding gap sites for our phylogenetic analyses. For the COI dataset, we used all the codon positions in our phylogenetic analyses.

**Figure 8.**
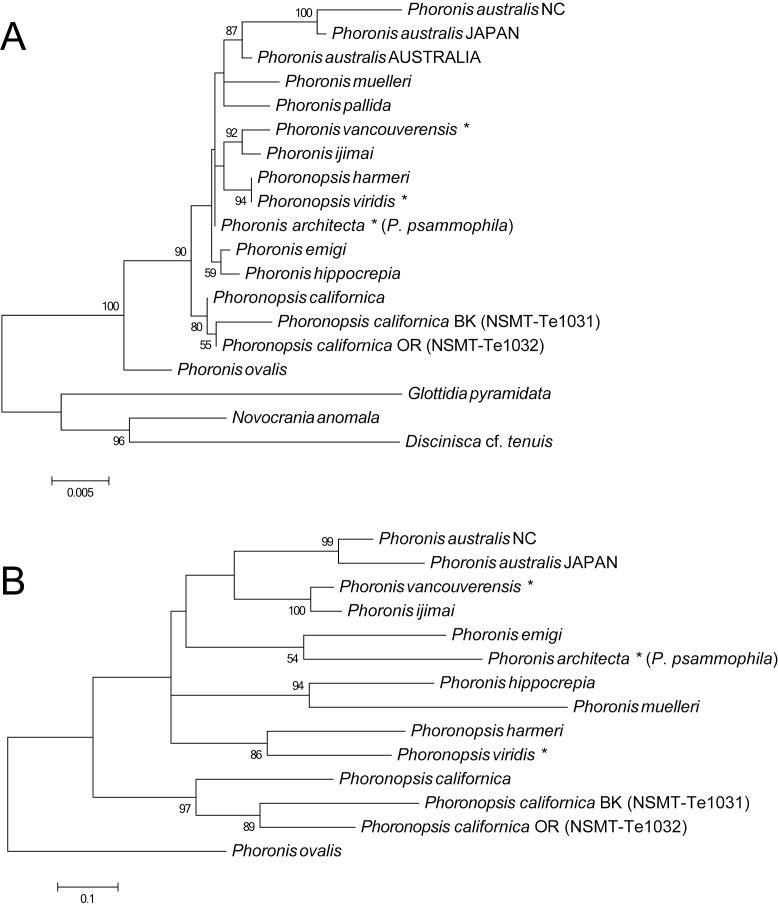
Maximum-Likelihood (ML) phylogenetic trees, based on 18S and COI data. **A** ML tree for 16 phoronid samples based on 18S data; three brachiopod species (*Novocraniaanomala*, Disciniscacf.tenuis and *Glottidiapyramidata*) are included as outgroup taxa; **B** ML tree for 14 phoronid samples based on COI data; the tree is rooted with *Phoronisovalis*. The scale bars represent branch length in substitutions per site. Nodal support values are presented as the bootstrap value; only values > 50% are shown. Specific names with an asterisk indicate the original names in GenBank before they were synonymised.

The 18S dataset comprised 1821 bp aligned sites, with 213 variable sites (1276 sites for final), for 19 ingroup taxa. In the resulting ML tree (Fig. 8A) (log *L* = −2833.08), most of the nodes are poorly resolved with low nodal support values. Although it is hard to reconstruct the phylogenetic relationships within the phylum, all of the same species appeared in a monophyletic clade with high nodal support: all *P.australis* appeared in a clade with 87, *P.ijimai* and nominal "*P.vancouverensis*" in a clade with 92 and *P.harmeri* and nominal "*P.viridis*" in a clade with 94. Two specimens of different colours of *P.californica* obtained in this study appeared in the same clade (55) which contains *P.californica* as a sister taxon, with relatively high nodal support (80).

The COI dataset comprised 705 aligned positions, with 264 variable sites (583 sites for final), for 14 ingroup taxa. In the resulting ML tree (Fig. 8B) (log *L* = −4137.94), not all nodes are resolved or well supported. Similar to the 18S tree above, all of the same species appear in a monophyletic clade with high nodal support: two *P.australis* from Japan and NC appeared in a clade with 99, *P.ijimai* and nominal "*P.vancouverensis*" in a clade with 100 and *P.harmeri* and nominal "*P.viridis*" in a clade with 86. Two specimens of *P.californica* obtained in this study appeared in a same clade (89) which contains *P.californica* as a sister taxon, with high nodal support (97).

## Discussion

This is the first report of the genus *Phoronopsis* in Japanese waters. According to the results, there are at least five species in two genera of phoronids in Japanese waters: *Phoronisijimai*, *P.australis*, *P.psammophila*, *P.emigi* and *Phoronopsiscalifornica*. Additionally, although without any description and figures, *Phoronispallida* was also listed from Tokyo Bay (Bailey-Brock and Emig 2000). Recently, nine phoronid species including unidentified species have been reported from the South China Sea (Temereva et al. 2016) and all known phoronid species are reported from the Indo-pacific waters; therefore, more intensive surveys at various localities may yield additional phoronid species in Japanese waters.

Our morphological and molecular results suggest both of our specimens in different colours are conspecific with *P.californica* that have previously been reported. In the present study, we follow the current idea that *P.viridis* is a junior synonym for *P.harmeri*. The Kimura 2-parameter (K2P) distance (Kimura 1980) amongst the different phoronid species for 583 bp of COI ranged from 0.164 (between *P.vancouverensis* and *P.australis* NC) to 0.31 (between the orange individual of *P.californica* collected in this study and *P.muelleri*). The K2P distance between our specimens and *P.californica* ranged from 0.171 (between the orange and black individuals collected in this study) to 0.195 (between *P.californica* and the black individual); the values are similar to or lower than the value of the intraspecific distance 0.194 between *P.harmeri* and *P.viridis* (Table 2). In contrast, the intraspecific distances amongst *Phoronis* ranged from 0.07 (between *P.ijimai* and *P.vancouverensis*) to 0.115 (between *P.australis* NC and *P.australis* JAPAN) in the same analysis; the difference of intraspecific K2P distance between phoronid genera may either represent differences in the evolutionally rate between genera or indicate a need for reconsideration of species classification within the *Phoronopsis*, including re-examination of synonymy.

Since the absence of any of hard structures and high range of the variation of external morphologies, phoronid taxonomy is largely depending on the internal morphologies, such as nephridia and the arrangement of the longitudinal muscles. Recent studies also revealed the intra- and inter-specific genetic distances; the results indicate a high range of the intra-specific morphological variation and the broad distribution of some species. However, the intensive genetic study on large numbers of specimens of nominal single species collected from different regions of the world still does not exist. Although most of the phoronid larvae (known as actinotrocha) are planktonic and have potentially broad dispersal ability, a high range of the intraspecific variation may suggest the presence of more undescribed species including cryptic species. The study on both morphological and genetic diversity of a single species, based on the specimens collected from extensive known localities, will be needed for further understanding of the global diversity of this phylum.

## Supplementary Material

XML Treatment for Phoronopsis californica

## Figures and Tables

**Table 1.** Taxa and the GenBank accession numbers for the sequences included in the phylogenetic analyses.

Species	COI	18S	Reference
*Phoronopsiscalifornica* OR	LC835680	LC835678	this study
*Phoronopsiscalifornica* BK	LC835679	LC835677	this study
*Phoronopsiscalifornica*	EU484463	EU334129	Santagata and Cohen (2009)
*Phoronopsisharmeri*	EU484464	EU334130	Santagata and Cohen (2009)
*Phoronopsisviridis*	EU484465	AF123308	Santagata and Cohen (2009)
*Phoronisaustralis* (New Caledonia)	EU484457	AF202111	COI - Santagata and Cohen (2009); 18S - Cohen (2000)
*Phoronisaustralis* (Japan)	EU484458	EU334122	Santagata and Cohen (2009)
*Phoronisaustralis* (Australia)	—	EU334123	Santagata and Cohen (2009)
*Phoronisijimai*	AB752304	AB752305	Hirose et al. (2014)
*Phoronisvancouverensis*	EU484462	AF202113	COI - Santagata and Cohen (2009); 18S - Cohen (2000)
*Phoronishippocrepia*	EU484459	AF202112	COI - Santagata and Cohen (2009); 18S - Cohen (2000)
*Phoronismuelleri*	EU484460	EU334125	Santagata and Cohen (2009)
*Phoronispallida*	—	EU334127	Santagata and Cohen (2009)
*Phoronisarchitecta*	AY368231.1	AF025946	COI - Helfenbein and Boore (2004)); 18S - Cohen et al. (1998)
*Phoronisemigi*	AB621915	AB621913	Hirose et al. (2014)
*Phoronisovalis*	EU484461	EU334126	Santagata and Cohen (2009)
*Novocraniaanomala*	—	AY842018	Cohen and Weydmann (2005)
Disciniscacf.tenuis	—	AY842020	Cohen and Weydmann (2005)
*Glottidiapyramidata*	—	U12647	Halanych et al. (1995)

**Table 2.** Pairwise genetic distances (K2P distances), based on 583 positions of COI sequences between two specimens of *P.californica* (*Ph.californica* OR and BK) and the other phoronid species. All of the intraspecific distances are also listed. The analysis involved 14 phoronid sequences. Specific names with an asterisk indicate the original names in GenBank before they were synonymised.

Species 1	Species 2	K2P Distance
*Phoronopsiscalifornica*	*Phoronopsiscalifornica* OR	0.184
	*Phoronopsiscalifornica* BK	0.195
*Phoronopsiscalifornica* OR	*Phoronopsiscalifornica* BK	0.171
*Phoronopsisharmeri*	*Phoronopsisviridis* *	0.194
*Phoronisijimai*	*Phoronisvancouverensis* *	0.070
*Phoronisaustralis* NC	*Phoronisaustralis* JAPAN	0.115
